# Supplementary material for: HIVprotI: an integrated web based platform for prediction and design of HIV proteins inhibitors
Source: J Cheminform. 2018 Mar 9;10:12. doi: 10.1186/s13321-018-0266-y (PMC5845081; doi:10.1186/s13321-018-0266-y)
Supplement: Supplementary file 2 — Additional file 2. Source code of HIVProtI web server. [file 13321_2018_266_MOESM2_ESM.zip › HIVprotI_Source-code/hivprotI/weka-3-6-6/doc/weka/core/SerializedObject.html]

SerializedObject


---


|  |  |  |
| --- | --- | --- |
| **Overview** \| **Package** \| **Class** \| **Tree** \| **Deprecated** \| **Index** \| **Help** \| **Weka's home** | |  |
| **PREV CLASS**   **NEXT CLASS** | **FRAMES**    **NO FRAMES**     **All Classes** |  |
| SUMMARY: NESTED \| FIELD \| CONSTR \| METHOD | DETAIL: FIELD \| CONSTR \| METHOD |  |


---


## weka.core Class SerializedObject

```
java.lang.Object
  weka.core.SerializedObject
```

**All Implemented Interfaces:**: java.io.Serializable, RevisionHandler

---

``` public class SerializedObject extends java.lang.Object implements java.io.Serializable, RevisionHandler ```

Class for storing an object in serialized form in memory. It can be used
to make deep copies of objects, and also allows compression to conserve
memory.

**Version:**
:   $Revision: 1.12 $

**Author:**
:   Richard Kirkby (rbk1@cs.waikato.ac.nz)

**See Also:**: Serialized Form

---

**Constructor Summary**

`SerializedObject(java.lang.Object toStore)`
  
          Creates a new serialized object (without compression).

`SerializedObject(java.lang.Object toStore,
boolean compress)`
  
          Creates a new serialized object.


| **Method Summary** | |
| --- | --- |
| `boolean` | `equals(java.lang.Object compareTo)` |
| `java.lang.Object` | `getObject()`             Returns a serialized object. |
| `java.lang.String` | `getRevision()`             Returns the revision string. |
| `int` | `hashCode()`             Returns a hashcode for this object. |

**Methods inherited from class java.lang.Object**

`getClass, notify, notifyAll, toString, wait, wait, wait`

**Constructor Detail**

### SerializedObject

```
public SerializedObject(java.lang.Object toStore)
                 throws java.lang.Exception
```

:   Creates a new serialized object (without compression).

    **Parameters:**: `toStore` - the object to store **Throws:**: `java.lang.Exception` - if the object couldn't be serialized

---


### SerializedObject

```
public SerializedObject(java.lang.Object toStore,
                        boolean compress)
                 throws java.lang.Exception
```

:   Creates a new serialized object.

    **Parameters:**: `toStore` - the object to store: `compress` - whether or not to use compression **Throws:**: `java.lang.Exception` - if the object couldn't be serialized


**Method Detail**

### equals

```
public final boolean equals(java.lang.Object compareTo)
```

:   **Overrides:**: `equals` in class `java.lang.Object`

---


### hashCode

```
public int hashCode()
```

:   Returns a hashcode for this object.

    :   **Overrides:**: `hashCode` in class `java.lang.Object`
    :   **Returns:**: the hashcode

---


### getObject

```
public java.lang.Object getObject()
```

:   Returns a serialized object. Uses org.python.util.PythonObjectInputStream
    for Jython objects (read
    here
    for more details).

    :   **Returns:**: the restored object **Throws:**: `java.lang.Exception` - if the object couldn't be restored

---


### getRevision

```
public java.lang.String getRevision()
```

:   Returns the revision string.

    :   **Specified by:**: `getRevision` in interface `RevisionHandler`
    :   **Returns:**: the revision


---


|  |  |  |
| --- | --- | --- |
| **Overview** \| **Package** \| **Class** \| **Tree** \| **Deprecated** \| **Index** \| **Help** \| **Weka's home** | |  |
| **PREV CLASS**   **NEXT CLASS** | **FRAMES**    **NO FRAMES**     **All Classes** |  |
| SUMMARY: NESTED \| FIELD \| CONSTR \| METHOD | DETAIL: FIELD \| CONSTR \| METHOD |  |


---
